# Loop-mediated isothermal amplification (LAMP) reaction as viable PCR substitute for diagnostic applications: a comparative analysis study of LAMP, conventional PCR, nested PCR (nPCR) and real-time PCR (qPCR) based on *Entamoeba histolytica* DNA derived from faecal sample

**DOI:** 10.1186/s12896-020-00629-8

**Published:** 2020-06-22

**Authors:** Phiaw Chong Foo, A. B. Nurul Najian, Nuramin A. Muhamad, Mariana Ahamad, Maizan Mohamed, Chan Yean Yean, Boon Huat Lim

**Affiliations:** 1 Acarology Unit, Infectious Disease Research Centre, Institute for Medical Research, Ministry of Health Malaysia, National Institutes of Health Complex, Bandar Setia Alam, 40170 Shah Alam, Selangor Malaysia; 2 Department of Medical Microbiology and Parasitology, School of Medical Sciences, Universiti Sains Malaysia, Health Campus, 16150 Kubang Kerian, Kelantan Malaysia; 3 Bacteriology Unit, Infectious Disease Research Centre, Institute for Medical Research, Ministry of Health Malaysia, National Institutes of Health Complex, Bandar Setia Alam, 40170 Shah Alam, Selangor Malaysia; 4 Institute for Research in Molecular Medicine, Universiti Sains Malaysia, Health Campus, 16150 Kubang Kerian, Kelantan Malaysia; 5 Faculty of Veterinary Medicine, Universiti Malaysia Kelantan, City Campus, Pengkalan Chepa, Locked bag 36, 16100 Kota Bharu, Kelantan Malaysia; 6 Hospital USM, Universiti Sains Malaysia, Health Campus, 16150 Kubang Kerian, Kelantan Malaysia; 7 School of Health Sciences, Universiti Sains Malaysia, Health Campus, 16150 Kubang Kerian, Kelantan Malaysia

**Keywords:** Loop-mediated isothermal amplification, Nested PCR, Real-time PCR, Lateral flow dipstick, Calcein-manganese visualization, LAMP analytical sensitivity

## Abstract

**Background:**

This study reports the analytical sensitivity and specificity of a Loop-mediated isothermal amplification (LAMP) and compares its amplification performance with conventional PCR, nested PCR (nPCR) and real-time PCR (qPCR). All the assays demonstrated in this study were developed based on Serine-rich *Entamoeba histolytica* protein (*SREHP*) gene as study model.

**Results:**

A set of *SREHP* gene specific LAMP primers were designed for the specific detection of *Entamoeba histolytica*. This set of primers recorded 100% specificity when it was evaluated against 3 medically important *Entamoeba* species and 75 other pathogenic microorganisms. These primers were later modified for conventional PCR, nPCR and qPCR applications. Besides, 3 different post-LAMP analyses including agarose gel electrophoresis, nucleic acid lateral flow immunoassay and calcein-manganese dye techniques were used to compare their limit of detection (LoD). One *E. histolytica* trophozoite was recorded as the LoD for all the 3 post-LAMP analysis methods when tested with *E. histolytica* DNA extracted from spiked stool samples. In contrast, none of the PCR method outperformed LAMP as both qPCR and nPCR recorded LoD of 100 trophozoites while the LoD of conventional PCR was 1000 trophozoites.

**Conclusions:**

The analytical sensitivity comparison among the conventional PCR, nPCR, qPCR and LAMP reveals that the LAMP outperformed the others in terms of LoD and amplification time. Hence, LAMP is a relevant alternative DNA-based amplification platform for sensitive and specific detection of pathogens.

## Background

DNA-based detection method has been widely used for diagnosis of infectious diseases due to the presence of specific DNA sequences in pathogens that can served as reliable detection biomarkers [1, 2]. This detection method is usually accompanied with amplification technology such as polymerase chain reaction (PCR), one of the most important scientific advances in molecular biology. PCR has established its molecular competency in term of detection sensitivity as it could amplify even a single gene copy [3]. Despite its popularity in disease diagnostic, PCR amplification possesses several inherent drawbacks such as primer mismatch due to high DNA similarity among species and low in copy number of specific gene for pathogen identification. These drawbacks have later paved the way for the emergences of several innovated PCR such as nested PCR (nPCR) and real-time PCR (qPCR).

The invention of nPCR was to increase the amplification efficacy in term of detection limit (LoD) and amplification specificity. PCR cycles which exceed 35 for production of larger quantity of product could cause generation of undesirable secondary amplicon [4]. nPCR could enhance the amplification sensitivity and priming specificity by incorporating 2 successive PCR reactions using 2 primer sets for a single gene target [5]. Despite addressing several advantages, nPCR was not commonly used for disease diagnostics due to its long turnaround time, and its two-step-procedure made it susceptible to amplicon contamination [6].

Real-time PCR (qPCR) technique on the other hand is well known with its concurrent detection and quantification of DNA. Its simultaneous amplicon analysis during amplification has significantly shortened the turnaround time by obviating post-amplification agarose gel electrophoresis. In spite of having comparable performance as nPCR [7–9], qPCR is machine-dependent which is often expensive and requires regular maintenance [10, 11].

Loop-mediated isothermal amplification (LAMP) technology was introduced in the year 2000 with the aim to improve nucleic acid amplification efficacy in term of sensitivity and specificity [12]. This technology has since incorporated into diagnostic assay development for detection of many medically important communicable diseases such as *Salmonella* Typhimurium [13], pathogenic *Leptospira* [14], *Enterococcus* spp. [15], and toxigenic *Vibrio cholerae* [16]. Incorporation of LAMP amplification in replacing PCR not only eliminated the needs of sophisticated thermal cycler, its DNA amplification efficiency beyond exponential had significantly shortened the amplification duration [12]. These established LAMP-based assays were reported to be highly specific and sensitive. Besides, the nature of LAMP that could synthesis DNA with auto-cycling strand displacement activity has effectively eliminated the need for costly thermocyclers and tedious technical optimisation of cycling conditions [17]. The efficacy of this technique used for disease diagnostic was further verified when development and assessment of assay using LAMP performed by Liang et al. [18], Rivera and Ong [19] and Singh et al. [20] for detection of *Entamoeba histolytica* alone have demonstrated outstanding compatibility.

Although previous studies have demonstrated the better amplification efficiency of LAMP as compared to PCR, there is yet a comprehensive report to evaluate the sensitivities among LAMP, conventional PCR, nPCR and qPCR. Therefore, the aim of this study is to compare the analytical sensitivity of LAMP assay with 3 different variants of PCR and concurrently determine the performance of LAMP using agarose gel electrophoresis, nucleic acid lateral flow immunoassay and calcein-manganese dye techniques as post-LAMP analyses. To ensure the equity of evaluation, all the assays primers were designed to bind on similar location of Serine-rich *Entamoeba histolytica* protein (*SREHP*) gene. The performance of these assays was evaluated using DNA isolated from stool samples spiked with *E. histolytica* trophozoites.

## Methods

### Reagents and apparatus

Goat anti-mouse IgG secondary antibody, streptavidin and fluorescein isothiocyanate (FITC) IgG1 monoclonal antibody used for development of nucleic acid lateral flow immunoassay or commonly named as lateral flow dipstick (LFD), were Pierce Thermo Fisher Scientific (Massachusetts, USA) products. The 40 nm colloidal gold solution used was from Kestrel Bio Sciences (Thailand), Western blocking reagent (WBR) was from Roche (Indianapolis, USA) and bovine serum albumin (BSA) was purchased from Amresco (Solon, USA). Betaine, mineral oil, sodium azide (NaN_3_), polyvinyl alcohol (PVA), polyvinylpyrrolidone (PVP), Triton X-100, Tween-20, sucrose, and other common chemicals were from Sigma (St. Louis, USA). All the chemicals and reagents used in this study were prepared using ultrapure water (>18MΩ) from a Millipore Milli-Q water purification system (Billerica, USA). Meanwhile, materials used for construction of LFD including cellulose fiber pads, glass fiber pads, and nitrocellulose membrane card HF135, were also Millipore products.

All labelled and non-labelled oligonucleotides were synthesised by Integrated DNA Technologies (Singapore). Recombinant *Taq* DNA polymerase (Thermo Fisher, USA) was used as polymerase enzyme for conventional PCR and nPCR amplification, and these reactions were carried out using Mastercycler nexus gradient thermocycler (Eppendorf, Germany). Meanwhile, qPCR was performed using QuantiFast SYBR Green PCR Kit (Qiagen, Germany) and the reaction was carried out using CFX96 Touch Real-Time PCR Detection System (Bio-Rad, California, USA). The LAMP *Bst* DNA polymerase was purchased from New England Biolabs (Massachusetts, USA) and the amplification were performed using Cole-Parmer chilling heating block (Illinois, USA). The conventional PCR, nPCR and LAMP amplicons were analysed using agarose gel electrophoresis system (Owl Separation Systems, USA) and visualised using Alpha Innotech ChemiImager 5500 UV illuminator and image capturing unit (California, USA). The LFD was lined with goat anti-mouse IgG secondary antibody and streptavidin manually, and were cut into strips using Matrix 2360 programmable strip cutter from Kinematic Automation (Twain Harte, USA). Calcein-manganese dye used for post-LAMP analysis was prepared using combination of calcein indicator (Merck, USA) and manganese (II) chloride (MnCl_2_) (Merck, USA).

### *Entamoeba* species and other microorganism strains

All the microorganism isolates used in this study are listed in Table 1. These isolates were from the Departamento de Medicina Experimental, Facultad de Medicina, Universidad Nacional Autónoma de México, Mexico; the London School of Hygiene and Tropical Medicine, London, UK; the Department of Medical Microbiology and Parasitology, School of Medical Sciences, Universiti Sains Malaysia, Malaysia; and the Institute for Medical Research, Malaysia. *E. histolytica HM-1*:IMSS was used as positive control while lyophilised *E. dispar* SAW760 and *E. moshkovskii* Laredo were used as negative controls in this study. DNA of *E. histolytica* was isolated from axenically grown *E. histolytica HM-1*:IMSS while DNA of *E. dispar* was isolated from lyophilised *E. dispar* SAW760 using Qiagen QIAamp DNA Stool extraction kit (Germany). Both organisms were received from the Departamento de Medicina Experimental, Facultad de Medicina, Universidad Nacional Autónoma de México, Mexico. Meanwhile, DNA of *E. moshkovskii* Laredo was given by the London School of Hygiene and Tropical Medicine, London, UK. DNA of other microorganisms were isolated from pure bacteria culture using NucleoSpin Tissue DNA Extraction kit (MACHEREY-NAGEL GmbH & Co. KG, Germany). These DNAs were used as negative controls for verification of conventional PCR, nPCR, qPCR and LAMP primers specificity.

**Table 1:** Reference organisms used in this study and analytical specificity evaluation results

**Strains**	**Serogroup or species**	**No. of isolates**	**Analytical specificity of primers**			
			**PCR**	**nPCR**	**qPCR**	**LAMP**
*Entamoeba* spp.	*Entamoeba histolytica HM-1*:IMSS	1	+	+	+	+
	*Entamoeba dispar* SAW760	1	–	–	–	–
	*Entamoeba moshkovskii* Laredo	1	–	–	–	–
Other enteric pathogens (*n* = 75)	*Acinetobacter baumannii*	1	–	–	–	–
	*Aeromonas hydrophila*	2	–	–	–	–
	*Bacillus subtilis*	1	–	–	–	–
	*Burkholderia cepacia*	1	–	–	–	–
	*Burkholderia pseudomallei*	1	–	–	–	–
	*Burkholderia thailandensis*	1	–	–	–	–
	*Citrobacter freundii*	1	–	–	–	–
	*Enterococcus faecalis*	1	–	–	–	–
	*Enterococcus faecium*	1	–	–	–	–
	*Enterococcus gallinarum*	1	–	–	–	–
	Enterohemorrhagic *Escherichia coli*	1	–	–	–	–
	Enteroinvasive *Escherichia coli*	1	–	–	–	–
	Enteropathogenic *Escherichia coli*	1	–	–	–	–
	Enterotoxigenic *Escherichia coli*	1	–	–	–	–
	*Escherichia coli*	1	–	–	–	–
	group A *Streptococcus*	1	–	–	–	–
	group B *Streptococcus*	1	–	–	–	–
	group F *Streptococcus*	1	–	–	–	–
	group G *Streptococcus*	1	–	–	–	–
	*Haemophilus influenza*	1	–	–	–	–
	*Klebsiella pneumoniae*	2	–	–	–	–
	*Leptospira biflexa* ser. Patoc	1	–	–	–	–
	*Leptospira interrogans* ser. Canicola	1	–	–	–	–
	*Leptospira interrogans* ser. Hebdomadis	1	–	–	–	–
	*Leptospira interrogans* ser. Pomona	1	–	–	–	–
	*Leptospira licerasiae* ser. Varillal	1	–	–	–	–
	*Listeria monocytogenes*	1	–	–	–	–
	*Salmonella enterica* ser. Agona	1	–	–	–	–
	*Salmonella enterica* ser. Albany	1	–	–	–	–
	*Salmonella enterica* ser. Bardo	1	–	–	–	–
	*Salmonella enterica* ser. Bordeaux	1	–	–	–	–
	*Salmonella enterica* ser. Braenderup	1	–	–	–	–
	*Salmonella enterica* ser. Emek	1	–	–	–	–
	*Salmonella enterica* ser. Enteritidis	1	–	–	–	–
	*Salmonella enterica* ser. Hadar	1	–	–	–	–
	*Salmonella enterica* ser. Heidelberg	1	–	–	–	–
	*Salmonella enterica* ser. Java	1	–	–	–	–
	*Salmonella enterica* ser. Kibi	1	–	–	–	–
	*Salmonella enterica* ser. Kissi	1	–	–	–	–
	*Salmonella enterica* ser. Newport	1	–	–	–	–
	*Salmonella enterica* ser. Oslo	1	–	–	–	–
	*Salmonella enterica* ser. Paratyphi A	2	–	–	–	–
	*Salmonella enterica* ser. Paratyphi B	2	–	–	–	–
	*Salmonella enterica* ser. Paratyphi C	1	–	–	–	–
	*Salmonella enterica* ser. Poona	1	–	–	–	–
	*Salmonella enterica* ser. Regent	1	–	–	–	–
	*Salmonella enterica* ser. Richmond	1	–	–	–	–
	*Salmonella enterica* ser. Tshiongwe	1	–	–	–	–
	*Salmonella enterica* ser. Typhi	2	–	–	–	–
	*Salmonella enterica* ser. Typhimurium	1	–	–	–	–
	*Salmonella enterica* ser. Uppsala	1	–	–	–	–
	*Salmonella enterica* ser. Virchow	1	–	–	–	–
	*Salmonella enterica* ser. Weltevreden	1	–	–	–	–
	*Shigella boydii*	2	–	–	–	–
	*Shigella dysenteriae*	2	–	–	–	–
	*Shigella flexneri*	2	–	–	–	–
	*Shigella sonnei*	2	–	–	–	–
	*Staphylococcus aureus*	1	–	–	–	–
	*Staphylococcus epidermidis*	1	–	–	–	–
	*Vibrio cincinnatiensis*	1	–	–	–	–
	*Vibrio fluvialis*	1	–	–	–	–
	*Vibrio mimicus*	1	–	–	–	–
	*Vibrio parahaemolyticus*	1	–	–	–	–
	*Vibrio vulnificus*	1	–	–	–	–
	*Yersinia enterocolitica*	2	–	–	–	–
Total		78				

+, positive result; −, negative result

### Primers design

**Fig. 1:**
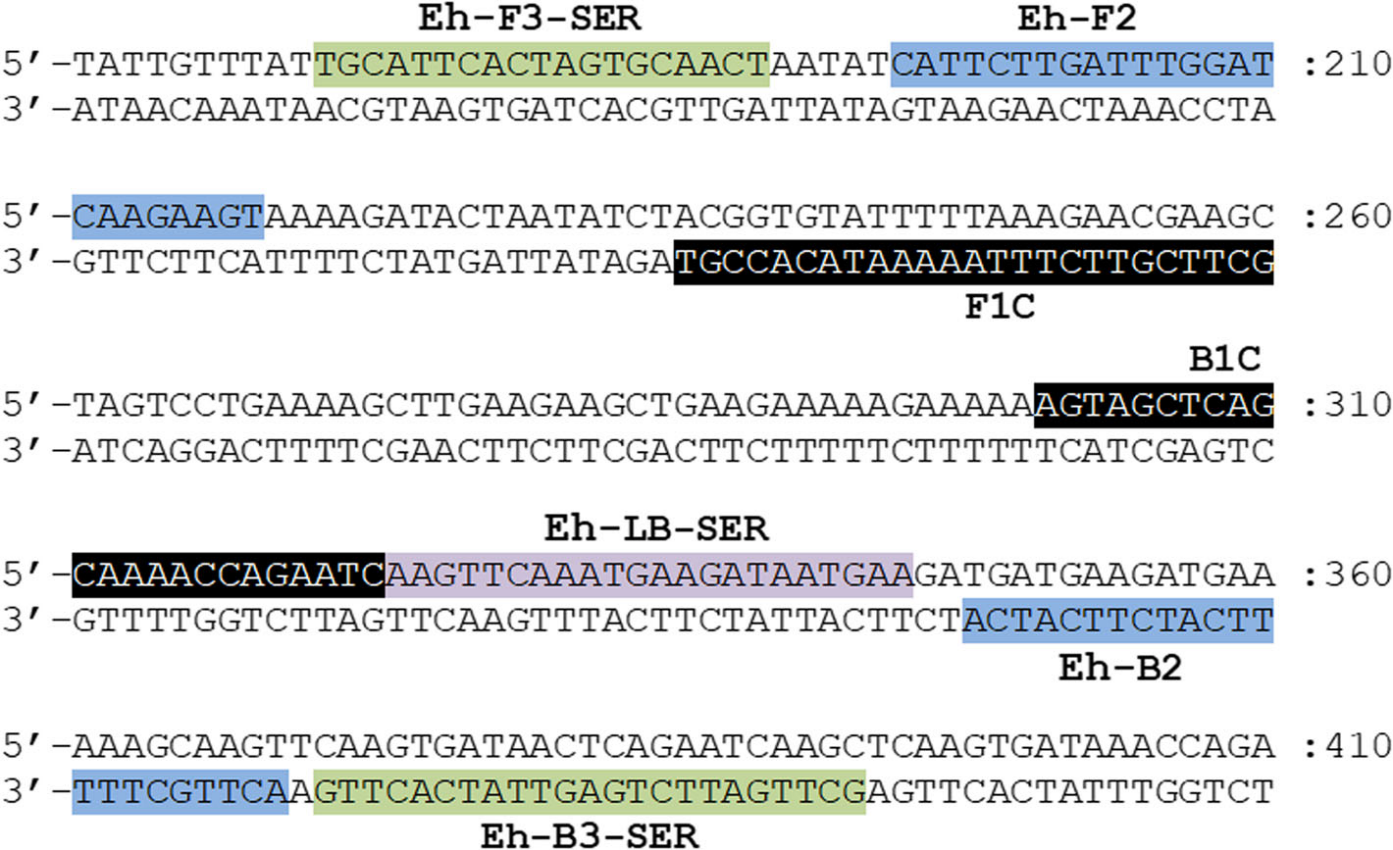
Primer regions on *SREHP* gene sequence (GenBank accession no. M80910.1). Eh-FIP-SER primer is formed with F1C and Eh-F2 while Eh-BIP-SER primer is formed with B1C and Eh-B2

*SREHP* gene was selected as the target gene for detection of *E. histolytica* in the present study due to its high specificity in both in silico (BLAST search) and empirical evaluation (PCR and LAMP amplification) compared to other *E. histolytica* genes. All the primers used in this study were designed based on the conserved region of the reported *SREHP* gene (GenBank accession no. M80910.1, M34438.1, XM_643162.2, AB253474.1, AK420158.1, AK420282.1, AK420358.1, AK420741.1). The primer set used for LAMP application was adopted from a previous study by Foo et al. [21] and the location of primers were shown in Fig. 1. Eh-F3-SER and Eh-B3-SER primers were used as outer primers for first round of nPCR amplification which could generate amplicon with size 223 bp. Meanwhile, the primer pair used for conventional PCR, second round of nPCR and qPCR amplification were adapted from F2 region of Eh-FIP-SER as forward primer and B2 region of Eh-BIP-SER as reverse primer; which could generate amplicon with size 175 bp. The primers used for LAMP amplification that coupled with LFD, particularly Eh-BIP-SER and Eh-LB-SER were subjected for modification using hapten-labelling at 5′ end of the oligonucleotide sequence. Fluorescein was labelled on the 5′ end of Eh-BIP-SER while Eh-LB-SER were labelled with biotin. All the sequence of primers used in this study were listed in Table 2. The specificity of these primers was verified empirically using DNA isolated from *E. histolytica*, *E. dispar*, *E. moshkovskii* Laredo and 75 other pathogens as shown in Table 1 before the comparison of analytical sensitivity among the assays was conducted.

**Table 2:** Oligonucleotide sequences used in this study

**Primers**	**Sequences (5′-3′)**	**Length (mer)**	**Product size**
**Loop-Mediated Isothermal Amplification**			
Eh-FIP-SER	GCTTCGTTCTTTAAAAATACACCGTCATTCTTGATTTGGATCAAGAAGT	49	–
Eh-BIP-SER	AGTAGCTCAGCAAAACCAGAATCACTTGCTTTTTCATCTTCATCA	45	
Eh-F3-SER	TGCATTCACTAGTGCAACT	19	
Eh-B3-SER	GCTTGATTCTGAGTTATCACTTG	23	
Eh-LB-SER	AAGTTCAAATGAAGATAATGAA	22	
**Conventional PCR & Real-Time PCR**			
Eh-F2	CATTCTTGATTTGGATCAAGAAGT	24	175 bp
Eh-B2	ACTTGCTTTTTCATCTTCATCA	22	
**Nested PCR**			
Eh-F3-SER	TGCATTCACTAGTGCAACT	19	223 bp
Eh-B3-SER	GCTTGATTCTGAGTTATCACTTG	23	
Eh-F2	CATTCTTGATTTGGATCAAGAAGT	24	175 bp
Eh-B2	ACTTGCTTTTTCATCTTCATCA	22	

### Formulation of LAMP, conventional PCR, nPCR and qPCR assays

#### LAMP

The outer primer to inner primer ratio was optimised and the concentration of primers were optimal with 2 μM of each forward inner primer (Eh-FIP-SER) and backward inner primer (Eh-BIP-SER), 0.167 μM of each forward outer primer (Eh-F3-SER) and backward outer primer (Eh-B3-SER), and 0.333 μM of backward loop primer (Eh-LB-SER). The concentrations of LAMP components such as dNTPs mix, betaine, MgSO4, and *Bst* DNA polymerase were optimised and determined empirically. The reaction was carried out with a final volume of 30 μL reaction mixture containing 1 × isothermal amplification buffer [20 mM of Tris-HCl (pH 8.8), 50 mM of KCl, 10 mM of (NH4)_2_SO_4_, 2 mM of MgSO_4_, 0.1% of Tween 20] (New England Biolabs, Massachusetts, USA), 0.6 mM of dNTP mix (Thermo Fisher Scientific, USA), 0.8 M of betaine (Sigma, Missouri, USA), supplementary 6 mM of MgSO_4_ (New England Biolabs, Massachusetts, USA), 16 U of *Bst* 2.0 WarmStart DNA polymerase (New England Biolabs, Massachusetts, USA) and 2 μL of DNA template. The reaction was carried out at 63 °C for 60 min followed by termination at 80 °C for 5 min. The LAMP product was subjected to agarose gel electrophoresis, LFD and calcein-manganese dye for post-LAMP analysis.

#### Conventional PCR

Eh-F2 and Eh-B2 primers with the concentration of 1 μM each were used for conventional PCR amplification. The amplification was carried out in a final volume of 20 μL containing 1 × PCR buffer, 2.5 mM of MgCl_2_ (Thermo Fischer Scientific, Massachusetts, USA), 0.16 mM of dNTPs mix (Thermo Fischer Scientific, Massachusetts, USA), 1 U of *Taq* DNA polymerase (Thermo Fischer Scientific, Massachusetts, USA) and 2 μL of DNA template. PCR reaction was performed with initial denaturation at 95 °C for 5 min, followed by 35 cycles of 95 °C for 30 s, 56 °C for 30 s and 72 °C for 30 s; and a final extension at 72 °C for 5 min. The product was subjected to gel electrophoresis in 2% agarose gel stained with GelStain dye (TransGen Biotech Co, Beijing), electrophoretically run under 100 V for 60 min followed by visualised using ChemiImage 5000 analyser.

#### nPCR

The first round of PCR was carried out with Eh-F3-SER and Eh-B3-SER primer pair with the concentration of 1 μM each. The amplification was carried out in a final volume of 20 μL containing 1 × PCR buffer, 2.5 mM of MgCl_2_ (Thermo Fischer Scientific, Massachusetts, USA), 0.16 mM of dNTPs mix (Thermo Fischer Scientific, Massachusetts, USA), 1 U of *Taq* DNA polymerase (Thermo Fischer Scientific, Massachusetts, USA) and 2 μL of DNA template. PCR reaction was performed with initial denaturation at 95 °C for 5 min, followed by 30 cycles of 95 °C for 30 s, 60 °C for 30 s and 72 °C for 30 s; and a final extension at 72 °C for 5 min. Meanwhile, the second round of PCR was carried out with Eh-F2 and Eh-B2 primer pair with the concentration of 1 μM each. The reagent composition used was similar to the first round PCR. The reaction was performed with initial denaturation at 95 °C for 5 min, followed by 35 cycles of 95 °C for 30 s, 56 °C for 30 s and 72 °C for 30 s; and a final extension at 72 °C for 5 min. The product was subjected to gel electrophoresis in 2% agarose gel stained with GelStain dye, electrophoretically run under 100 V for 60 min then visualised using ChemiImage 5000 analyser.

#### qPCR

Similar to conventional PCR and second round reaction of nPCR, the qPCR reaction was carried out with Eh-F2 and Eh-B2 primer pair with the concentration of 1 μM each. The amplification was carried out in a final volume of 25 μL containing 1 × QuantiFast SYBR Green PCR Master Mix and 2 μL of DNA template. The reaction was performed with thermal cycling condition of initial denaturation at 95 °C for 5 min, followed by 40 cycles of 95 °C for 20 s and 56 °C for 30 s. Melting curve analysis was performed at a slow increase from 65 °C to 95 °C with a speed of 0.5 °C per 5 s. Baseline threshold for the post-amplification analysis was set at 50 relative fluorescence units (RFU) and any quantitation cycle (Cq) value below or equal to 38 is considered positive.

### Preparation of post-LAMP analysis

#### Construction of LFD

**Fig. 2:**
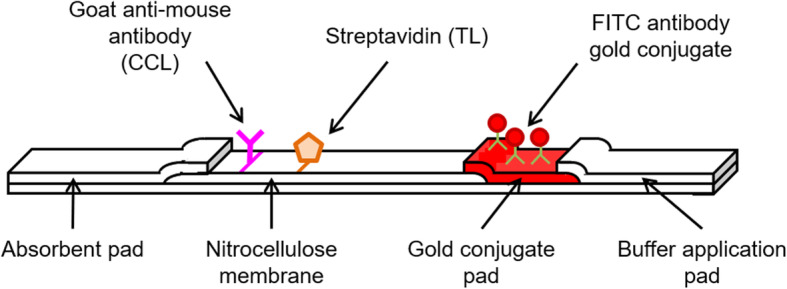
Schematic diagram of lateral flow dipstick strip

Gold nanoparticle (GNP) was used as signal generator for LFD in this study. The bio-conjugation of GNPs with FITC IgG1 monoclonal antibody and the preparation of LFD was carried out as described by Foo et al. [21] with some modification. The LFD strip with size of 5 mm × 77 mm composed with buffer application pad, gold conjugate pad, nitrocellulose membrane and an absorbent pad as shown in Fig. 2. The LFD was affixed with 1 μg goat anti-mouse IgG secondary antibody as chromatography control line (CCL) and 2 μg streptavidin as test line (TL) followed by block the uncoated nitrocellulose surface with blocking buffer [mixture of 0.2% WBR, 0.05% triton X-100 and 2 mM phosphate buffer (PB)]. The gold conjugate pad was made with functionalised conjugated GNPs suspension [5 OD_522_ gold conjugate suspended in 2 mM PB containing 20% (w/v) sucrose, 0.01% (v/v) PVA and 0.01% (v/v) Tween-20] in dry-reagent format as described by Foo et al. [21].

#### Preparation of calcein-manganese dye

Calcein-manganese dye was made of 500 μM calcein that dissolved in dimethyl sulfoxide (Merck, USA) and 10 mM MnCl_2_ that dissolved in nuclease-free water. Only 1 μL of calcein-manganese dye required for every reaction.

#### Principle of LFD

**Fig. 3:**
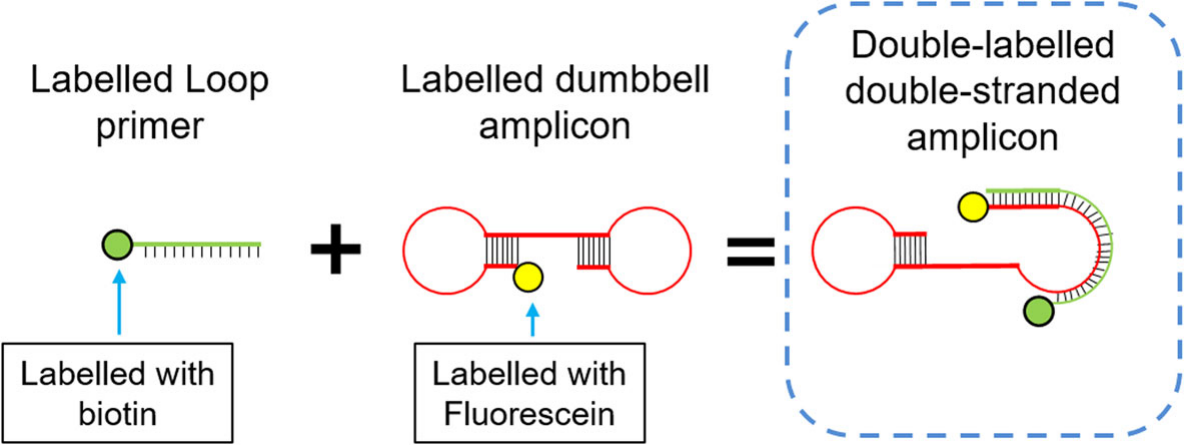
Schematic illustration of the formation of double-labelled amplicon (LAMP product) works as the analyte for LFD detection

The LFD was formulated to specifically recognise its double-labelled double-stranded DNA amplicon through the binding of the biotin labelled on the 5′ end of amplicons to the streptavidin on nitrocellulose membrane of LFD. Test line required amplified double-labelled double-stranded amplicons as the connection bridge to generate signal that represent the presence/absence of the target DNA. The double-labelled double-stranded amplicons (LAMP product) for TL were labelled with FAM at 5′ end synthesised by inner primers whereas another 5′ end that synthesised by loop primer were labelled with biotin Fig. 3. Streptavidin on TL formed protein-ligand binding with biotin on doubled-labelled amplicons. Meanwhile, CCL affixed with goat anti-mouse IgG secondary antibody formed protein–ligand affinity binding with mouse monoclonal FITC IgG1 antibody which conjugated on gold nanoparticles.

**Fig. 4:**
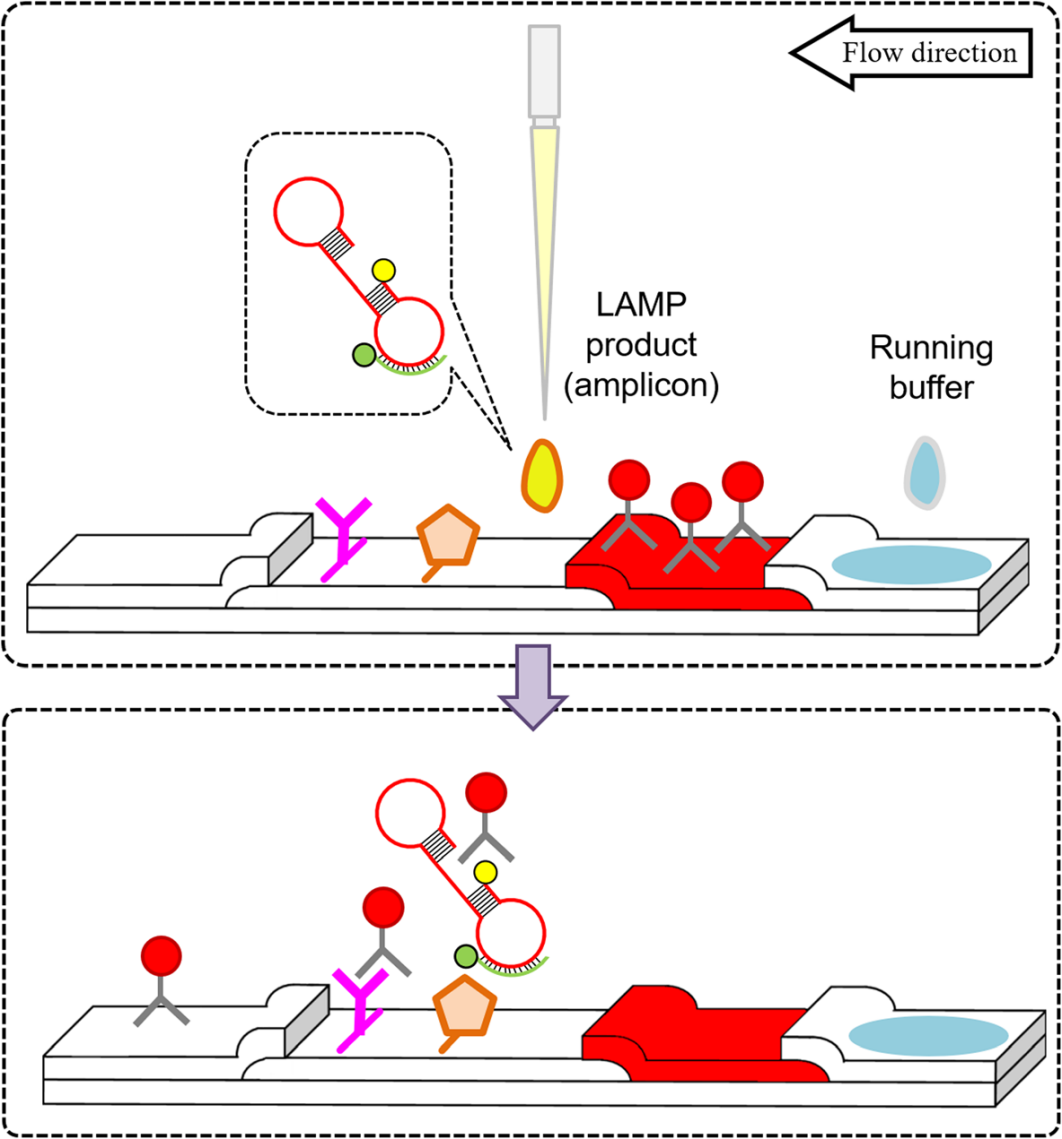
Schematic illustration of the principle of LFD. The illustration shows the LAMP product is immobilised on the membrane by streptavidin, followed with signal generation by conjugated GNPs that bind on fluorescein

Figure 4 illustrated a schematic principle of the LAMP product captured by streptavidin on detection pad of LFD. Streptavidin affixed on the detection region immobilised the amplicons through protein-ligand bonding formed with the biotin labelled on the amplicons. The double-stranded amplicons were formed by the LAMP inner primer sequences and loop primer sequences. Therefore, the other 5′ end of amplicon was presented with FAM that bound with goat anti-mouse antibody conjugated on gold nanoparticles. The presence of pinkish red line on the detection region showed the completion of hybridisation sandwich among the conjugated GNPs, LAMP product and streptavidin whereby interpreted as positive result.

### Post-LAMP analysis

Post-LAMP analysis was carried out in 3 different techniques namely agarose gel electrophoresis, LFD and calcein-manganese dye. LAMP product was subjected to gel electrophoresis in 2.5% agarose gel stained with GelStain dye, electrophoretically run under 100 V for 80 min followed by visualised using ChemiImage 5000 analyser. The present of ladder-like bands pattern on the agarose gel indicated positive amplification.

Detection of LAMP product using LFD was similar to a previous study [21]. The amplified product with an amount of 4 μL was mixed with 16 μL of running buffer [1 × PBS and 1% (v/v) Tween-20] and the mixture was dropped onto the detection region. The LFD was then placed vertically followed by dipping the buffer application pad into 250 μL of running buffer. The result could be visualised with unaided eyes within 10 to 15 min on the LFD nitrocellulose membrane. The formation of red dotted line on TL indicated positive result while the absent of TL indicated negative result. CCL on the LFD served as procedural and operational control for every LAMP product analysis. The line formation on CCL indicated effectiveness of signal generator, the functionalised GNPs while the absent of CCL indicated false negative result.

Analysis of LAMP product using calcein-manganese dye was carried out by mixing 1 μL of calcein-manganese dye into the 30 μL of LAMP reagent mix before amplification. With the aid of UV light, tube that turned fluorescent green was considered positive while negative amplification remained as dim green.

### Analytical specificity of assays

The analytical specificity of the primers used for LAMP, conventional PCR, nPCR and qPCR application were determined using DNA isolated from *E. histolytica*, *E. dispar*, *E. moshkovskii* Laredo and 75 other pathogens as listed in Table 1. To ensure its specificity was comprehensively investigated, this study was conducted separately on LAMP, conventional PCR, nPCR and qPCR.

### Evaluation of assays performance

All the 4 amplification assays were optimised in this study. The performance of the assays was evaluated based on their analytical sensitivity in term of LoD using 10-fold diluted *E. histolytica* trophozoites. The diluted trophozoites in a range of 10^6^ to 10^− 4^ trophozoites were spiked into 200 mg of stool samples and left for 1 h at ambient temperature prior to DNA isolation. Extracted DNAs from the spiked stool samples in a range of 10^6^ to 10^− 3^ trophozoites were used for PCR while LoD for LAMP amplification was determined using trophozoites range up to 10^− 4^. All the evaluation tests were performed in triplicate.

## Results

### Development and optimisation of LAMP assay

**Fig. 5:**
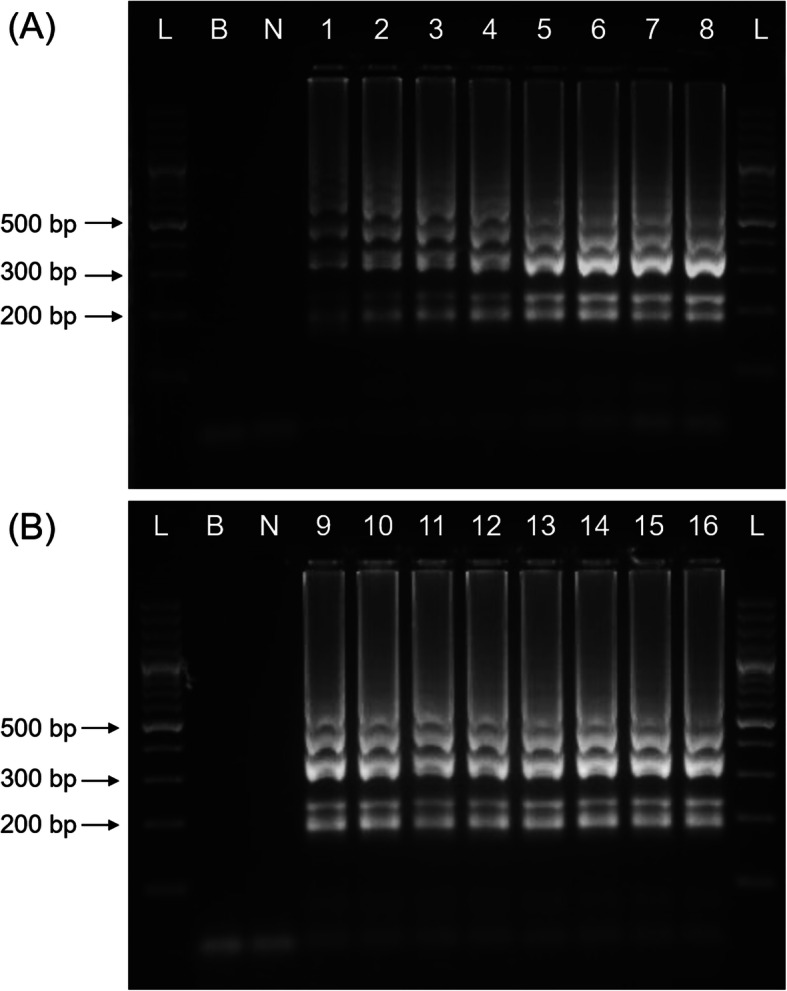
Optimisation of LAMP (**a**) inner primers concentration and (**b**) outer primers concentration. L, 100 bp DNA ladder; B, blank (no DNA template control); N, negative control; 1–16, primer concentrations in μM: lane 1, 0.33; lane 2, 0.67; lane 3, 1.0; lane 4,1.33; lane 5, 1.67; lane 6, 2.0; lane 7, 2.33; lane 8, 2.67; lane 9, 0.083; lane 10, 0.10; lane 11, 0.117; lane 12, 0.133; lane 13, 0.167; lane 14, 0.25; lane 15, 0.33; lane 16, 0.67. The selected optimum inner primers concentration was 2.0 μM while 0.167 μM was selected as optimum outer primers concentration

Investigation on LAMP primer ratio was prioritised before other LAMP components were optimised as incompatible ratio for inner and outer LAMP primers may affect the amplification sensitivity [22]. The outer primers to inner primers ratio was determined using two separated experiments, namely inner primers concentration optimisation and outer primers concentration optimisation. The optimisation for inner primers concentration was conducted by altering the concentrations of inner primers from 0.33 to 2.67 μM with the outer primers kept at 0.167 μM. On the other hand, the concentration of outer primers was optimised in a range of 0.083 to 0.67 μM with the inner primers kept at 1.33 μM. The optimal inner primers concentration was found to be 2.0 μM (Fig. 5a), while the optimal outer primers concentration was recorded as 0.167 μM (Fig. 5b).

**Fig. 6:**
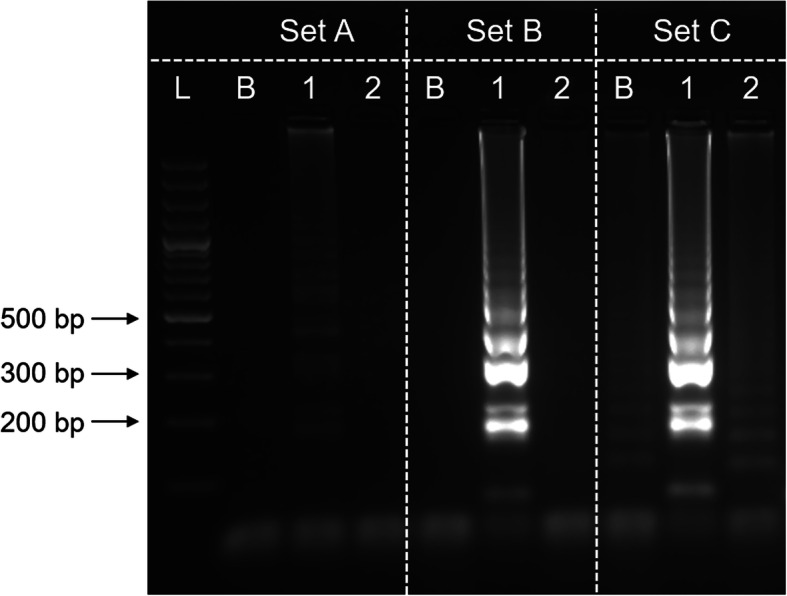
Effect of betaine in LAMP amplification. L, 100 bp plus DNA ladder; B, blank (no DNA template control); 1, *E. histolytica*; 2, *E. dispar*. The concentration of betaine used were: set A, 1.6 M; set B, 0.8 M; set C, 0.4 M. The selected optimum betaine concentration was 0.8 M

LAMP components were optimised to ensure the efficiency of amplification. Parameters including incubation temperature, concentrations of betaine, MgSO_4_, dNTP mix, and *Bst* DNA polymerase were empirically determined. Optimisation of LAMP parameters did not significantly improve the efficiency of amplification. However, study on the betaine concentration showed excess of betaine could deter the efficiency of amplification. Figure 6 showed the outcome of LAMP amplification performed with 3 different concentrations (1.6 M, 0.8 M and 0.4 M). LAMP reaction performed with lower betaine concentration (0.4 M) could tolerate false positive result while excess betaine (1.6 M) could deter amplification efficiency. LAMP amplification conducted without DNA template and with non-*E. histolytica* DNA could generate false positive result when 0.4 M betaine was incorporated into the reaction. Although amplification with 1.6 M betaine does not generate false positive result on negative control and non-target control, the product intensity of positive control on agarose gel was faint. Therefore, 0.8 M was selected as the optimum concentration for betaine. The optimised 30 μL of LAMP reaction mix was concluded as optimal with 1 × LAMP amplification buffer, 0.8 M of betaine, 6 mM of MgSO_4_, 0.6 mM of dNTP mix and 16 U of *Bst* DNA polymerase with incubation temperature of 63 °C.

### Analytical performances of LAMP, conventional PCR, nPCR and qPCR assays

#### Analytical specificity

The primers analytical specificity for LAMP, conventional PCR, nPCR and qPCR respective application using DNA isolated from *E. histolytica*, *E. dispar*, *E. moshkovskii* Laredo and 75 other pathogens revealed 100% specificity. The analytical specificity of the primers used for their respective amplifications is summarised in Table 1.

#### Analytical sensitivity

Detection limit of the 4 assays were investigated using DNAs isolated from 10-fold diluted trophozoites that were spiked into stool samples. Those trophozoites were spiked into stool samples obtained from healthy individuals to simulate isolation of DNA from stool of infected individual. The extracted DNAs in a range of 10^6^ to 10^− 3^ trophozoites were used for PCR applications while LAMP amplification was tested using up to 10^− 4^ trophozoites due to its excellent amplification efficacy.

**Fig. 7:**
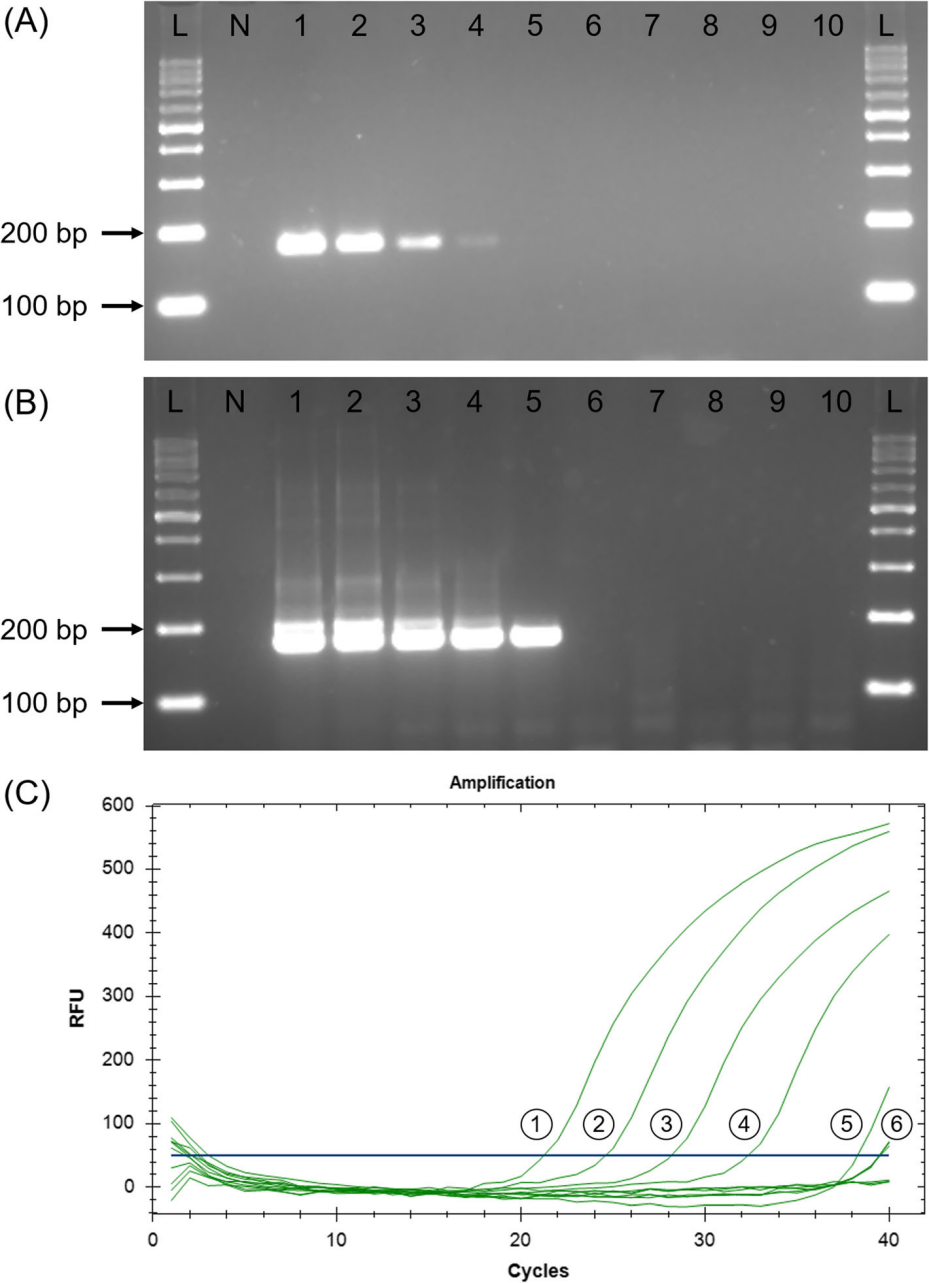
Spiked stool analytical sensitivity on (**a**) conventional PCR, (**b**) nPCR and (**c**) qPCR using 10-fold dilutions of *E. histolytica* trophozoites. L, 100 bp DNA ladder; N, negative control; 1–10, 10-fold dilution of trophozoites: lane 1, 10^6^; lane 2, 10^5^; lane 3, 10^4^; lane 4, 10^3^; lane 5, 10^2^; lane 6, 10; lane 7, 1; lane 8, 0.1; lane 9, 10^− 2^; lane 10, 10^− 3^. PCR could detect up to 1000 trophozoites while nPCR and qPCR could detect up to 100 trophozoites

**Fig. 8:**
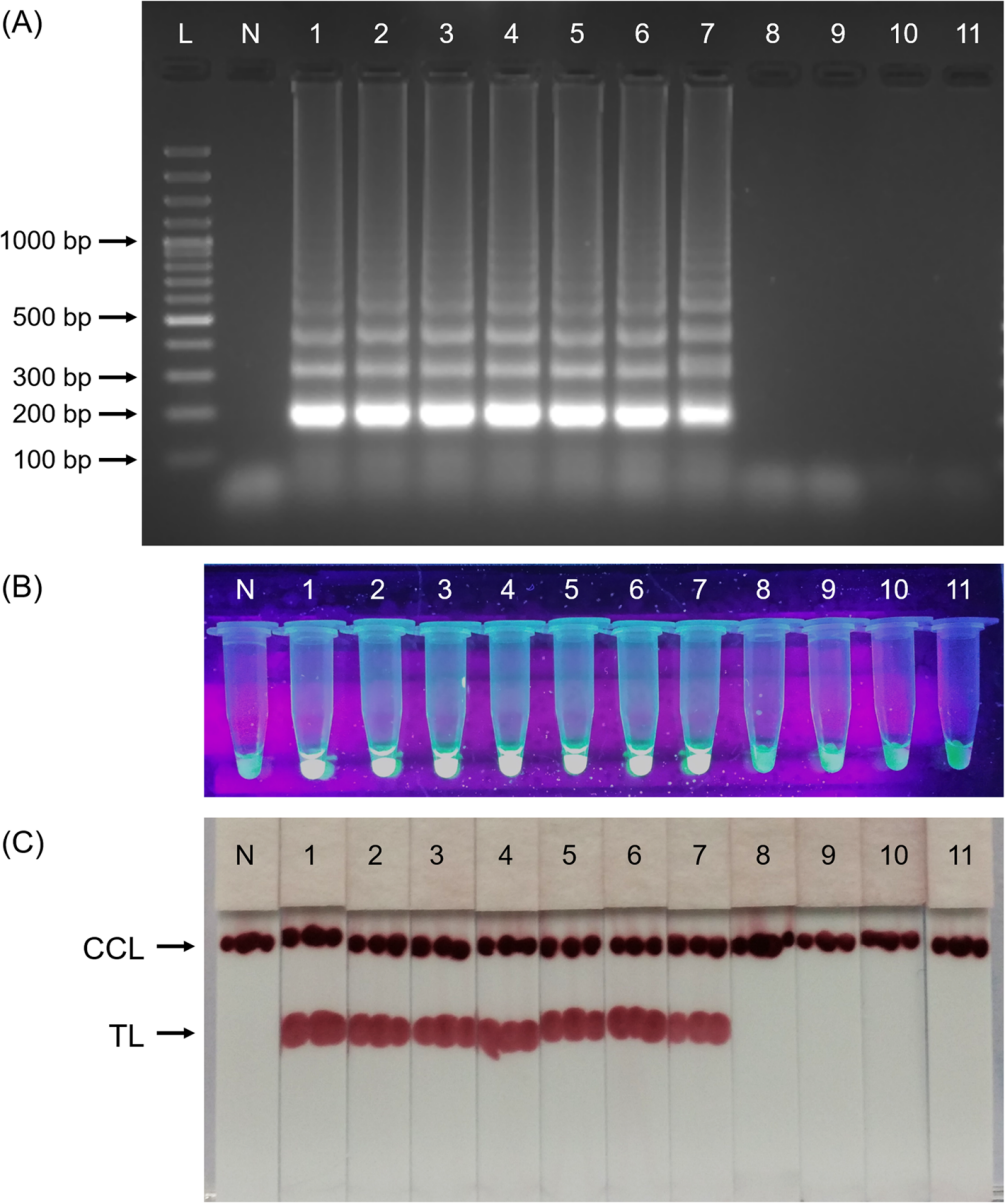
Spiked stool analytical sensitivity on LAMP using 10-fold dilutions of *E. histolytica* trophozoites and analysed using (**a**) agarose gel electrophoresis, (**b**) calcein-manganese dye and (**c**) LFD. L, 100 bp DNA ladder; N, negative control; 1–10, 10-fold dilution of trophozoites: lane 1, 10^6^; lane 2, 10^5^; lane 3, 10^4^; lane 4, 10^3^; lane 5, 10^2^; lane 6, 10; lane 7, 1; lane 8, 0.1; lane 9, 10^− 2^; lane 10, 10^− 3^; lane 11, 10^− 4^. All the 3 post-LAMP analyses recorded 1 trophozoite as LoD

The LoD for conventional PCR was found to be 1000 trophozoites as shown in Fig. 7a while nPCR recorded 10-fold more sensitive LoD as amplicon was still observable when tested with DNA extracted from 100 trophozoites (Fig. 7b). As only curve goes above 50 RFU with ≤38 Cq value will be interpreted as positive (see methods), the analytical sensitivity of qPCR was found to be similar to nPCR, wherein was recorded as 100 trophozoites (Fig. 7c). The analytical sensitivity of the LAMP assay conducted using 3 different post-LAMP analyses recorded similar LoD in which agarose gel electrophoresis (Fig. 8a), calcein-manganese dye (Fig. 8b) and LFD (Fig. 8c) techniques similarly recorded 1 trophozoite as LoD.

## Discussion

PCR has always been the DNA-based detection method of choice for identification of medically important pathogen. As for amoebiasis, this method has been endorsed by WHO for detection of *E. histolytica*. This technology has been reported to detect *E. histolytica* in various clinical specimens and could differentiate it from other morphologically indistinguishable non-pathogenic *Entamoeba* species [23, 24]. However, the presence of amplification inhibitors has hindered PCR application for detection of *E. histolytica* in faecal samples. Examples of the amplification inhibitors include bilirubins, bile salts, heme and carbohydrates and such molecule complexes could be co-extracted along with the pathogen DNA in stool samples [25].

LAMP amplification that uses single temperature to amplify target gene was described to be more robust and less affected by inhibitory agents found in clinical samples [26]. This finding was further verified when Engku Nur Syafirah et al. [16] found reduction of amplification sensitivity on PCR while LAMP could retain its efficacy when tested with DNA isolated from spiked stool samples. The robustness of LAMP has encouraged several developments of diagnostic assays. In regards to diagnosis of amoebiasis, Liang et al. [18], Rivera and Ong [19], Singh et al. [20] and Foo et al. [21] had shown the potential of LAMP in terms of efficiency and applicability for detection of the pathogen. This study was conducted to further compare the LAMP amplification performance with conventional PCR, nPCR and qPCR.

Empirical analysis on the designed primers using cross-amplification analysis and primer concentration optimisation were conducted to maximise the amplification efficacy. During LAMP amplification, the inner primer F2 and B2 regions would first bind and polymerise their complementary nucleotide sequences before adherence of outer primers F3 and B3 followed by executing strand displacement activity. This mechanism showed the LAMP inner primers played an important role in determining the specificity of LAMP amplification. Therefore, conventional PCR, and qPCR amplifications were solely conducted using F2 and B2 regions of LAMP inner primers while nPCR was conducted with additional LAMP outer primers. Primers specificity verification showed generation of expected product sizes on agarose gel electrophoresis while their cross-amplification with other *Entamoeba* spp. and non-*Entamoeba* DNAs also showed expected specificities.

Cross-amplification was conducted on LAMP primer set using LAMP amplification and the results obtained showed expected outcomes. During the LAMP assay development, the concentration of inner and outer primer was investigated to avoid disproportion primers ratio that could affect the amplification efficiency [22]. This optimisation revealed increment of inner primers concentration could significantly increase the amplification yield while the involvement of outer primers in LAMP amplification did not alter the amplicon yield. Besides, the LAMP outer primers that was only involved in displacing the inner primer to allow the formation of dumbbell-like DNA structure was devoid with F3 and B3 regions. Therefore, the strategy of having fluorescein labelled on backward inner primer and biotin on loop primer was relevant and applicable.

During the development of LAMP assay in this study, betaine was found to be an essential component that could enhance the specificity of LAMP amplification. The optimisation study conducted with lower betaine concentrations (0.4 M) has facilitated the formation of false negative results. However, excess increment of betaine (1.6 M) could distort the amplification efficiency and reduce the assay sensitivity. Therefore, betaine optimisation is crucial in every LAMP assay development to eliminate unspecific amplification and yet retain its amplification efficiency.

The application of direct visual identification on LAMP product using intercalating fluorescent dye has been reported to be sensitive and specific [27, 28]. An in-house calcein-manganese dye was used as post-LAMP analysis for direct visual identification on LAMP product in this study. This fluorescent indicator made use of pyrophosphate, a LAMP amplification by-product, to generate fluorescent signal by allowing manganese ion to form complex molecule with pyrophosphate and leaving free calcein to irradiate the fluorescence under UV light. Lateral flow dipstick-based assay is a feasible diagnostic platform because it is simple to perform, produce rapid visual result, amendable for mass-production, relatively cheap to produce, does not require equipment for result interpretation and portable [3]. The in-house dry-reagent LFD could detect target nucleic acid by capturing the hapten labelled amplicon and generated visual signal using functionalised GNPs [21]. Incorporation of dry-reagent technology on LFD for efficacy preservation and stability enhancement has expanded its usability and made it operator friendly. Therefore, these 2 methods were selected as alternative post-LAMP analyses for sensitivity comparison with agarose gel electrophoresis.

This study revealed the sensitivity of PCR amplification platform was not comparable to LAMP regardless of its amplification and amplicon analysis innovations. Although the addition of amplification cycles as demonstrated by nPCR and qPCR could promote yield increment, excessive amplification cycles in a single reaction would risk faulty priming that could generate false positive result [4]. Besides, the enhancement still lacked sensitivity as LAMP could detect DNA with concentration of 100-fold lower compared to nPCR and qPCR. Meanwhile, the similar LoD recorded by the 3 LAMP post-amplification analysis demonstrated reproducibility of LAMP amplification. The analytical sensitivity consistency despite being coupled with different post-amplification analysis methods justified the compatibility and robustness of LAMP as an alternative nucleic acid amplification test to PCR. The strength of LFD and calcein-manganese dye used during post-LAMP analysis also upheld the potential of LAMP in becoming an equipment-free diagnostic assay that complied to ASSURED principle. This principle which stands for Affordable, Sensitive, Specific, User-friendly, Rapid and robust, Equipment-free and Deliverable was deemed relevant and in demand for better disease control in the developing countries with low resource setting [29].

Despite LAMP outstanding performance, the technology was less favourable for multiplexing, which involved tolerating amplification of multiple targets in a single reaction. Moreover, the typical ladder-like pattern of LAMP product, produced by mixture of dumbbell-like structures with various stem length are indistinguishable under gel electrophoresis and intercalating fluorescent dye. This setback was resolved by Najian et al. [14] who developed a duplex LAMP assay with the aid of LFD; followed by the expansion of the technique to a triplex LAMP assay by Foo et al. [21]. The incorporation of internal amplification control into these assays to rule out false negative result caused by amplification inhibitors has further enhanced LAMP feasibility as an alternative molecular technique for detection of pathogens.

## Conclusion

The analytical sensitivity comparison among the conventional PCR, nPCR, qPCR and LAMP reveals that LAMP outperformed the rest in terms of LoD and amplification time. Meanwhile, all the 3 post-LAMP analyses appeared to be similar in detection sensitivities. Hence, LAMP is a relevant alternative DNA-based amplification platform for sensitive and specific detection of pathogens.

## Data Availability

All data generated or analysed during this study are included in this published article.

## Abbreviations

BSA: Bovine serum albumin
*Bst*: *Bacillus stearothermophilus*
CCL: Chromatography control line
dNTPs: Deoxynucleoside triphosphates
FITC: Fluorescein isothiocyanate
GNP: Gold nanoparticle
LAMP: Loop-mediated isothermal amplification
LFD: Lateral flow dipstick
LoD: Limit of detection
nPCR: Nested polymerase chain reaction
PVA: Polyvinyl alcohol
PVP: Polyvinylpyrrolidone
qPCR: Real-time polymerase chain reaction
RFU: Relative fluorescence units
*SREHP*: Serine-rich *Entamoeba histolytica* protein
*Taq*: *Thermus aquaticus*
TL: Test line
WBR: Western blocking reagent

## References

[1] Chang D, Tram K, Li B, Feng Q, Shen Z, Lee CH, Salena BJ, Li Y (2017). Detection of DNA amplicons of polymerase chain reaction using litmus test. Sci Rep.

[2] Law JW, Ab Mutalib NS, Chan KG, Lee LH (2014). Rapid methods for the detection of foodborne bacterial pathogens: principles, applications, advantages and limitations. Front Microbiol.

[3] Yager P, Domingo GJ, Gerdes J (2008). Point-of-care diagnostics for global health. Annu Rev Biomed Eng.

[4] Lorenz TC (2012). Polymerase chain reaction: basic protocol plus troubleshooting and optimization strategies. J Vis Exp.

[5] Foo PC, Chan YY, See Too WC, Tan ZN, Wong WK, Lalitha P, Lim BH (2012). Development of a thermostabilized, one-step, nested, tetraplex PCR assay for simultaneous identification and differentiation of *Entamoeba* species, *Entamoeba histolytica* and *Entamoeba dispar* from stool samples. J Med Microbiol.

[6] Khlif M, Mary C, Sellami H, Sellami A, Dumon H, Ayadi A, Ranque S (2009). Evaluation of nested and real-time PCR assays in the diagnosis of candidaemia. Clin Microbiol Infect.

[7] Kawada J, Kimura H, Ito Y, Hoshino Y, Tanaka-Kitajima N, Ando Y, Futamura M, Morishima T (2004). Comparison of real-time and nested PCR assays for detection of herpes simplex virus DNA. Microbiol Immunol.

[8] Kim HS, Kim DM, Neupane GP, Lee YM, Yang NW, Jang SJ, Jung SI, Park KH, Park HR, Lee CS (2008). Comparison of conventional, nested, and real-time PCR assays for rapid and accurate detection of Vibrio vulnificus. J Clin Microbiol.

[9] Wells B, Shaw H, Innocent G, Guido S, Hotchkiss E, Parigi M, Opsteegh M, Green J, Gillespie S, Innes EA (2015). Molecular detection of toxoplasma gondii in water samples from Scotland and a comparison between the 529bp real-time PCR and ITS1 nested PCR. Water Res.

[10] Jiang HX, Liang ZZ, Ma YH, Kong DM, Hong ZY (2016). G-quadruplex fluorescent probe-mediated real-time rolling circle amplification strategy for highly sensitive microRNA detection. Anal Chim Acta.

[11] Niemz A, Ferguson TM, Boyle DS (2011). Point-of-care nucleic acid testing for infectious diseases. Trends Biotechnol.

[12] Notomi T, Okayama H, Masubuchi H, Yonekawa T, Watanabe K, Amino N, Hase T (2000). Loop-mediated isothermal amplification of DNA. Nucleic Acids Res.

[13] Techathuvanan C, Draughon FA, D'Souza DH (2010). Loop-mediated isothermal amplification (LAMP) for the rapid and sensitive detection of Salmonella Typhimurium from pork. J Food Sci.

[14] Najian ABN (2016). Syafirah EAREN, Ismail N, Mohamed M, yean CY: development of multiplex loop mediated isothermal amplification (m-LAMP) label-based gold nanoparticles lateral flow dipstick biosensor for detection of pathogenic *Leptospira*. Anal Chim Acta.

[15] Martzy R, Kolm C, Brunner K, Mach RL, Krska R, Sinkovec H, Sommer R, Farnleitner AH, Reischer GH (2017). A loop-mediated isothermal amplification (LAMP) assay for the rapid detection of Enterococcus spp. in water. Water Res.

[16] Engku Nur Syafirah EAR, Nurul Najian AB, Foo PC, Mohd Ali MR, Mohamed M, Yean CY (2018). An ambient temperature stable and ready-to-use loop-mediated isothermal amplification assay for detection of toxigenic Vibrio cholerae in outbreak settings. Acta Trop.

[17] Buchan BW, Ledeboer NA (2014). Emerging technologies for the clinical microbiology laboratory. Clin Microbiol Rev.

[18] Liang SY, Chan YH, Hsia KT, Lee JL, Kuo MC, Hwa KY, Chan CW, Chiang TY, Chen JS, Wu FT (2009). Development of loop-mediated isothermal amplification assay for detection of *Entamoeba histolytica*. J Clin Microbiol.

[19] Rivera WL, Ong VA (2013). Development of loop-mediated isothermal amplification for rapid detection of *Entamoeba histolytica*. Asian Pac J Trop Med.

[20] Singh P, Mirdha BR, Ahuja V, Singh S (2013). Loop-mediated isothermal amplification (LAMP) assay for rapid detection of *Entamoeba histolytica* in amoebic liver abscess. World J Microbiol Biotechnol.

[21] Foo PC, Chan YY, Mohamed M, Wong WK, Nurul Najian AB, Lim BH (2017). Development of a thermostabilised triplex LAMP assay with dry-reagent four target lateral flow dipstick for detection of Entamoeba histolytica and non-pathogenic Entamoeba spp. Anal Chim Acta.

[22] Yeh HY, Shoemaker CA, Klesius PH (2005). Evaluation of a loop-mediated isothermal amplification method for rapid detection of channel catfish *Ictalurus punctatus* important bacterial pathogen *Edwardsiella ictaluri*. J Microbiol Methods.

[23] Fotedar R, Stark D, Beebe N, Marriott D, Ellis J, Harkness J (2007). Laboratory diagnostic techniques for *Entamoeba* species. Clin Microbiol Rev.

[24] Parija SC, Mandal J, Ponnambath DK (2014). Laboratory methods of identification of *Entamoeba histolytica* and its differentiation from look-alike *Entamoeba* spp. Tropical Parasitol.

[25] Holland JL, Louie L, Simor AE, Louie M (2000). PCR detection of *Escherichia coli* O157:H7 directly from stools: evaluation of commercial extraction methods for purifying fecal DNA. J Clin Microbiol.

[26] Kaneko H, Kawana T, Fukushima E, Suzutani T (2007). Tolerance of loop-mediated isothermal amplification to a culture medium and biological substances. J Biochem Biophys Methods.

[27] Tao ZY, Zhou HY, Xia H, Xu S, Zhu HW, Culleton RL, Han ET, Lu F, Fang Q, Gu YP (2011). Adaptation of a visualized loop-mediated isothermal amplification technique for field detection of plasmodium vivax infection. Parasit Vectors.

[28] Zhou D, Guo J, Xu L, Gao S, Lin Q, Wu Q, Wu L, Que Y (2014). Establishment and application of a loop-mediated isothermal amplification (LAMP) system for detection of cry1Ac transgenic sugarcane. Sci Rep.

[29] Wu G, Zaman MH (2012). Low-cost tools for diagnosing and monitoring HIV infection in low-resource settings. Bull World Health Organ.

